# No Effect of Exercise Intensity on Appetite in Highly-Trained Endurance Women

**DOI:** 10.3390/nu8040223

**Published:** 2016-04-18

**Authors:** Stephanie M. Howe, Taryn M. Hand, D. Enette Larson-Meyer, Kathleen J. Austin, Brenda M. Alexander, Melinda M. Manore

**Affiliations:** 1 Nutrition and Exercise Science, School of Biological and Population Health Sciences, Oregon State University, Corvallis, OR 97331, USA; stephaniemariehowe@gmail.com (S.M.H.); tarynmhand@gmail.com (T.M.H.); 2 Department of Family and Consumer Sciences, University of Wyoming, Laramie, WY 82071, USA; enette@uwyo.edu (D.E.L.-M.); KathyAus@uwyo.edu (K.J.A.); BAlex@uwyo.edu (B.M.A.)

**Keywords:** high-intensity exercise, moderate-intensity exercise, female athletes, appetite-hormones

## Abstract

In endurance-trained men, an acute bout of exercise is shown to suppress post-exercise appetite, yet limited research has examined this response in women. The purpose of this study was to investigate the effect of exercise intensity on appetite and gut hormone responses in endurance-trained women. Highly-trained women (*n* = 15, 18–40 years, 58.4 ± 6.4 kg, VO_2MAX_ = 55.2 ± 4.3 mL/kg/min) completed isocaloric bouts (500 kcals or 2093 kJ) of moderate-intensity (MIE, 60% VO_2MAX_) and high-intensity (HIE, 85% VO_2MAX_) treadmill running at the same time of day, following a similar 48-h diet/exercise period, and at least 1-week apart. Blood was drawn pre-exercise (baseline), immediately post-exercise and every 20-min for the next 60-min. Plasma concentrations of acylated ghrelin, PYY_3–36_, GLP-1 and subjective appetite ratings via visual analog scale (VAS) were assessed at each time point. Acylated ghrelin decreased (*p* = 0.014) and PYY_3–36_ and GLP-1 increased (*p* = 0.036, *p* < 0.0001) immediately post-exercise, indicating appetite suppression. VAS ratings of hunger and desire to eat decreased immediately post-exercise (*p* = 0.0012, *p* = 0.0031, respectively), also indicating appetite suppression. There were no differences between exercise intensities for appetite hormones or VAS. Similar to males, post-exercise appetite regulatory hormones were altered toward suppression in highly-trained women and independent of energy cost of exercise. Results are important for female athletes striving to optimize nutrition for endurance performance.

## 1. Introduction

The importance of exercise as part of a healthy lifestyle, both for prevention of chronic diseases and weight management, is well established. Based on the 2008 Physical Activity Guidelines for Americans, the public health recommendations for physical activity (PA) include daily exercise for at least 150 min/week to promote and maintain health [1]. Additional health benefits of regular PA include reduction of risk factors for chronic diseases, such as coronary heart disease, type II diabetes, and stroke, increased energy expenditure, improved metabolic function, and maintenance of lean tissue [1,2]. However, the role of exercise in the regulation of appetite and energy intake is still being explored [3].

Appetite and food intake are complex behaviors, influenced by the integration of numerous hormonal and neural signals in the hypothalamus [4]. Gut hormones, including ghrelin, peptide YY (PYY), and glucagon-like peptide 1 (GLP-1) are potent regulators of appetite influenced by exercise [4,5,6]; however, the magnitude and duration of this effect has yet to be determined.

Recent studies in highly-trained men have shown that an acute bout of exercise can affect appetite by altering appetite-regulating hormones in the direction of appetite suppression [7,8,9,10,11]. However, only one study has examined the impact of an acute bout of exercise on appetite-regulating hormones in highly-trained women, with inconsistent results compared to highly-trained men [12].

Examination of exercise intensity on appetite-regulating hormones has only been examined in highly-trained men, with results showing an intensity-dependent influence [10,13]. In these studies, high-intensity exercise had a greater influence on appetite suppression compared to moderate-intensity exercise. However, no study has investigated the impact of different exercise intensities on appetite-regulating hormones in highly-trained women.

Thus, the purpose of this study was to investigate the influence of exercise intensity on objective and subjective measures of appetite in highly-trained women. We hypothesized that high-intensity exercise (HIE; 85% VO_2MAX_) would have a greater appetite suppression effect on appetite-regulatory hormones compared to moderate-intensity exercise (MIE; 60% VO_2MAX_). A secondary aim was to determine the impact of intensity exercise on subjective appetite ratings. We also hypothesized that HIE would suppress post-exercise appetite ratings to a greater degree than MIE, while controlling energy expenditure during exercise.

## 2. Materials and Methods

### 2.1. Participants and Recruitment

Highly-trained endurance female athletes (*n* = 15; 18–40 years) were recruited for this randomized crossover design study. Eligibility criteria were as follows: (1) Good health and considered “low risk” for cardiovascular disease as indicated by the American College of Sports Medicine criteria for exercise testing and prescription [14]; (2) Minimum of 5-day/week of endurance exercise of ~30–60 min/session; (3) VO_2MAX_ ≥ 50 mL·kg^−1^∙min^−1^ ± 5%; (3) Not pregnant or lactating; and (4) Stable body mass (<2.3 kg weight change) over the past 6 months. The study was approved by the University Institutional Review Board and all participants were informed prior to signed consent.

### 2.2. Baseline Testing

VO_2MAX_ was determined using a standardized treadmill test with 4-mins at a moderate intensity, based on individual average training and race pace, then speed and grade increased every minute until exhaustion (~8–12-min). Oxygen consumption (VO_2_) and carbon dioxide production (VCO_2_) were measured breath-by-breath using a metabolic cart (ParvoMedics TrueOne 2400, Sandy, UT, USA). Heart rate (HR) was monitored and recorded from a Polar HR monitor and rating of perceived exertion (RPE) using the Borg scale was recorded in the last 10 s of each stage. Two of the following four criteria were required for a test to be considered maximal: (1) a plateau in VO_2_ despite increasing workload; (2) Respiratory Exchange Ratio (RER) ≥1.10; (3) maximal HR within 10 beats of age predicted max (max HR = (208 − (0.7 × age in year)) [15]; (4) RPE ≥ 17. Subject demographics, including height, body mass, and body composition via skinfold calipers, were measured and recorded.

### 2.3. Experimental Protocol

The study consisted of two randomized experimental exercise trials separated by at least 7 days: (1) MIE trial at 60% VO_2MAX_ and, (2) HIE trial at 85% VO_2MAX_ (See Table 1). For both trials, participants ran on the treadmill until an exercise energy expenditure (EEE) of 500 kcal (2093 kJ) was achieved. The speed and duration for each intensity was calculated using VO_2MAX_ test data and verified by measuring VO_2_ (±5%) during the first 5-min of the test and HR and RPE throughout the trial. Water was not consumed during the trials, but participants were allowed to consume water *ad libitum* after the exercise trials. During both trials, the room temperature was maintained at a temperature between 18 and 20 °C.

To standardize diet and PA before each trial, participants self-recorded 48-h weighted food and PA logs and refrained from exercise 24-h prior to testing. The 48-h before the second visit they were asked to mimic the first visit as closely as possible, including testing at the same time of day, and maintaining food intake and PA patterns. Diet and PA logs were reviewed before each trial to check for compliance, with all participants found to be compliant.

### 2.4. Exercise Trials, Gut Hormones and Appetite Assessments

On the exercise trial day, participants arrived to the laboratory at least 4-h post-prandial. On the first visit, each subject was randomly assigned to either the MIE or HIE exercise trial. Before beginning the exercise trial, a baseline blood sample was taken from an antecubital vein. All participants completed the trials at the calculated duration and intensity and at the same time of day.

Immediately post-exercise, participants were transferred to a reclining chair and blood was drawn (post-exercise time 0-min), and every 20-min over the next 60-min (post-exercise 20, 40, 60-min). Previous research has shown that gut appetite hormones peak immediately post-exercise and return to baseline within 30–60-min post-exercise [10,12]. Subjective measures of appetite were also completed at baseline prior to the exercise trial and post-exercise prior to each blood draw (0, 20, 40, 60-min). During the 60-min post-exercise recovery period, participants rested in the recliner and movement was minimal. Participants consumed water on an ad libitum basis. Following the final blood draw, participants were provided with food and drink.

### 2.5. Subjective Appetite Assessment

The visual analog scale (VAS) (100 mm scale) was used to record hunger, satiety, fullness, and desire to eat [16]. During the exercise trials, VAS was recorded prior to each blood draw at the following time points: baseline, immediately post-exercise (0-min), and 20, 40, and 60-min post-exercise.

### 2.6. Hormone Analysis

All blood samples were analyzed for plasma concentrations of acylated ghrelin, PYY_3–36_, and GLP-1. Blood samples were collected into EDTA-treated pre-chilled tubes. Samples collected for acylated ghrelin analysis contained 100 μL of 200 mM·AEBSF with 200 μL of 1 N·HCL added after centrifuging for each mL of plasma. Samples collected for PYY_3–36_ analysis were treated with 150 μL of aprotinin and 40 μL of DPP-IV. All samples were cold centrifuged (2–8 °C) for 10 min at 3500 rpm and immediately frozen at −80 °C for later analysis. Samples were batch analyzed after completion of the study in duplicate by radioimmunoassay using commercially available kits specific for human (Millipore, St. Charles, MO, USA).

### 2.7. Statistical Analysis

Sample size (*n* = 15) was based on previous research examining the influence of exercise intensity on appetite hormones in healthy men by Ueda and colleagues [10]. Their sample size of 10 participants was sufficient to detect significant differences in appetite regulating hormones, including PYY_3–36_, and GLP-1 post-exercise *vs.* control trial (no exercise) (α = 0.05). Broom *et al.*, [7] reported statistically significant acylated ghrelin during exercise compared to rest in 9 males running 60-min at 72% VO_2MAX_ (α = 0.001).

A mixed model analysis was used to examine the impact of exercise intensity (60% (MIE) *vs.* 85% (HIE) VO_2MAX_) on appetite-regulatory hormones (acylated ghrelin, PYY, GLP-1) and VAS responses over time (baseline (pre-exercise), post-exercise (0-min), 20, 40 and 60-min). The PROC MIXED procedure in SAS (version 9.3 for Windows, Cary, NC, USA) was used to analyze the data. Exercise intensity and time were modeled as fixed effects. The covariance structure was modeled by treating the ten combinations of time and exercise intensity for each subject as repeated measures. This process was repeated for the VAS data examining changes in hunger, satisfied, fullness and desire-to-eat, over time for each exercise intensity. Where significant effects were found, post-hoc analyses were performed using the Bonferroni correction for multiple comparisons.

We also determined total response over time for the appetite-regulatory hormones and appetite ratings (VAS) by calculating area under the curve (AUC) using the trapezoid method (GraphPad Prism Version 6.0 for Mac OS X, GraphPad Software, San Diego, CA USA. Our AUC calculation included all area above and below baseline (net area). Paired t-tests were used to examine if any differences existed between MIE and HIE trials. The overall statistical significance was accepted at the 5% level. Results are presented as Mean ± standard deviation (SD).

## 3. Results

### 3.1. Baseline Characteristics

Participants (*n* = 15) were highly-trained endurance female athletes competing in sports such as running, cycling, Nordic skiing, and triathlon. On average, participants trained approximately 13 h/week (range 7–25 h/week) including endurance sports and cross training (e.g., swimming, yoga, and strength training.) (See Table 2).

### 3.2. Moderate and High Intensity Exercise Trials

Participants ran on the treadmill 45.7 ± 10.8 min and 33.6 ± 5.6 min on average for MIE and HIE trials, respectively (See Table 3).

### 3.3. Appetite Hormones

**Figure 1:**
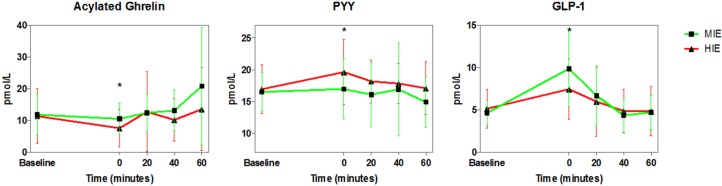
Appetite Hormone Responses to Moderate-Intensity Exercise (MIE) and High-Intensity Exercise (HIE) in Highly-Trained Female Endurance Athletes. Each exercise trial = 500 kcal (2093 kJ). * Significantly different from baseline (*p* < 0.0125).

**Figure 2:**
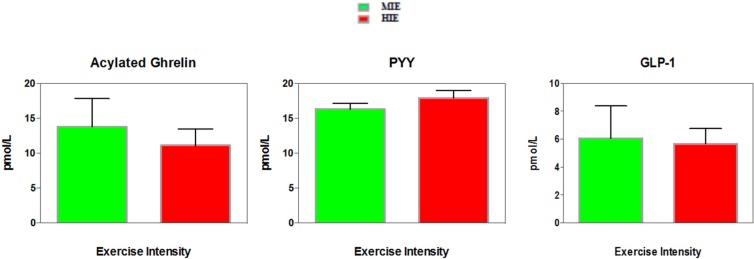
Average Area Under the Curve (AUC) Appetite Hormones Response in Moderate-Intensity Exercise (MIE) and High-Intensity Exercise (HIE) Trials in Highly-Trained Female Endurance Athletes.

There were no differences between the two exercise intensity trials for acylated ghrelin (*p* = 0.07), PYY (*p* = 0.14), or GLP-1 (*p* = 0.40). However, there was a significant main effect for time due to exercise for all three hormones. Immediately post-exercise (time point 0), acylated ghrelin was significantly lower (*p* = 0.014), and PYY and GLP-1 were significantly higher (*p* = 0.02, *p* = 0.04, respectively) compared to baseline. By 60-min post-exercise, acylated ghrelin, PYY, and GLP-1 were not significantly different from baseline (*p* = 0.06, *p* = 0.44, *p* = 0.75, respectively; see Figure 1 and Figure 2).

There were no differences between exercise trials for AUC for acylated ghrelin (*p* = 0.055) and PYY (*p* = 0.54). For GLP-1, the MIE trial showed a slightly higher AUC compared to the HIE trial (*p* = 0.04, uncorrected), however once corrected, the difference was no longer significant (see Figure 1 and Figure 2).

### 3.4. Appetite Ratings

**Figure 3:**
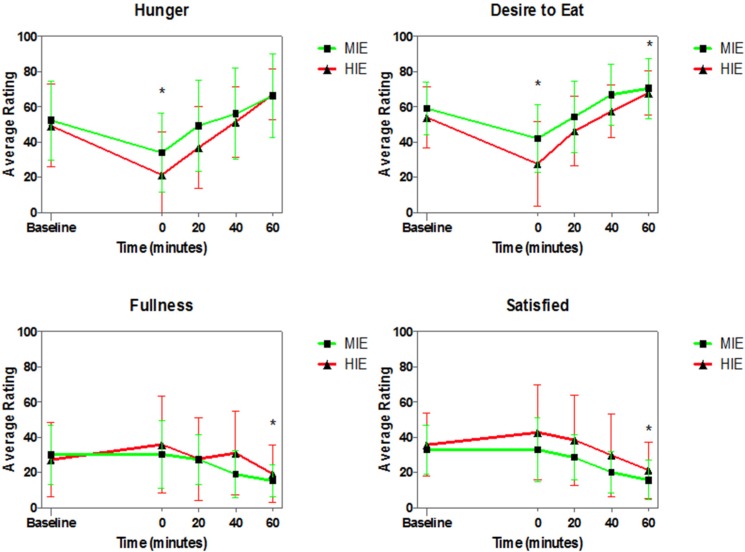
Appetite Ratings (Visual Analog Scale) in Moderate-Intensity Exercise (MIE) and High-Intensity Exercise (HIE) in Highly-Trained Female Endurance Athletes. Each trial expended 500 kcal (2093 kJ). * Significantly different from baseline (*p* < 0.0125).

Similar to the appetite-hormones, there was a significant main effect of time for all appetite ratings. Ratings of hunger and desire to eat were significantly lower immediately post-exercise (*p* = 0.0012, *p* = 0.0031, respectively), while ratings of satiety and fullness were significantly lower at 60-min post-exercise (*p* = 0.0005, *p* = 0.0006, respectively). Additionally, ratings of desire to eat were significantly higher 60-min post-exercise (*p* = 0.0028, See Figure 3).

**Figure 4:**
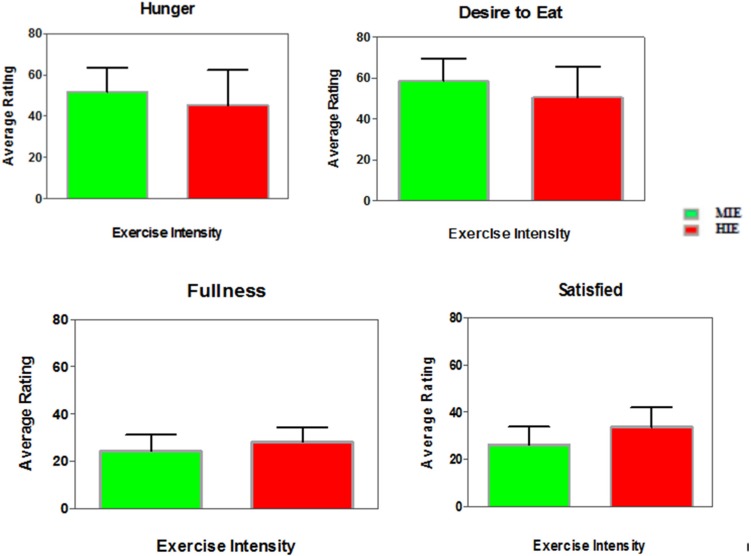
Average Area Under the Curve (AUC) Appetite Ratings in Moderate-Intensity Exercise (MIE) and High-Intensity Exercise (HIE) Trials in Highly-Trained Female Endurance Athletes.

The AUC analysis showed no differences between the MIE and HIE trials for ratings of hunger (*p* = 0.15), satisfied (*p* = 0.06), or desire to eat (*p* = 0.12). The AUC for fullness was significantly lower after the MIE trial compared to HIE trial (*p* = 0.03, uncorrected) (See Figure 4).

## 4. Discussion

To our knowledge, this was the first study to evaluate the impact of two different exercise intensities on appetite in highly-trained women. Our primary aim was to determine the impact of exercise intensity on appetite-regulating hormones (acylated ghrelin, PYY_3–36_, GLP-1) in highly-trained women (VO_2MAX_ ≥ 50 mL/kg/min ± 5%). Overall, there were no differences between exercise intensity (HIE *vs.* MIE) on appetite-regulatory hormones; however, there were significant time effects due to exercise. Immediately post-exercise, there were significant increases in circulating anorexogenic peptides, PYY_3–36_ and GLP-1, and a decrease in the orexigenic peptide acylated ghrelin. Thus, an acute bout of endurance exercise resulted in significant changes in circulating appetite-regulating hormones in the direction of suppression.

Our second aim was to determine the impact of MIE and HIE on subjective appetite response in highly-trained women. We hypothesized that HIE would suppress post-exercise appetite responses to a greater degree than MIE. Overall, we saw no differences in AUC between the two exercise trials, except for fullness where there was a trend for a lower response after HIE *vs.* MIE.

### 4.1. Sex Differences and Exercise Intensity

The research examining whether men and women exhibit similar hormonal responses to exercise is limited with available studies demonstrating equivocal results. Hagobian and colleagues [17] found that sedentary, overweight men had significantly lower post-exercise appetite ratings (desire to eat, hunger, and how much food you can eat) while in women exercise had no effect on the same appetite ratings. Further, the women had a 25% increase in post-exercise acylated ghrelin while there was no difference for the men. In a subsequent study with healthy, normal weight habitually active participants, there were no differences were found between men and women for subjective and objective appetite measures [18]. Only one study has measured appetite-regulating hormones in exercise-trained females following an acute bout of running [12]. Similar to our study, PYY and GLP-1 increased following 60-min of treadmill running at 70% VO_2MAX_, while in contrast, acylated ghrelin increased immediately post-exercise (VO_2MAX_ = 49.7 mL/kg/min). The current study is the only study to evaluate the impact of two exercise intensities on appetite in highly-trained women.

Research examining the impact of exercise on appetite-regulating hormones has used a variety of different exercise conditions including different exercise modes, durations, and intensities. These studies have examined exercise intensities ranging from 45% to 85% VO_2MAX_ in highly-trained individuals [7,8,9,10,11,12,13,19,20,21,22,23,24,25,26,27,28,29,30], yet few studies have compared two exercise intensities within the same study [10,31]. In highly-trained males, only two studies have compared the impact of different intensities (50%–85% VO_2MAX_) on appetite-regulating hormones [10,31]. Collectively, these studies have found that HIE (70%–85% VO_2MAX_) resulted in greater appetite suppression compared to MIE (50%–60% VO_2MAX_). However, this has not been investigated in highly-trained women (VO_2MAX_ ≥ 40 mL/kg/min).

### 4.2. Exercise and Appetite-Regulating Hormones

Our results are consistent with other studies that have investigated the impact of an acute bout of endurance exercise on appetite in highly-trained men (VO_2MAX_ ≥ 45.9 mL/kg/min) [7,8,9,10,11,13,19,20,21,23,24,25,26,32]. In these studies, cycling and running at intensities ranging from 50% to 70% VO_2MAX_ for 45–90 min resulted in significant changes of circulating acylated ghrelin, PYY, and GLP-1 that would suggest appetite blunting. Only one study, however, has investigated the impact of acute exercise on appetite-regulating hormones in highly-trained women [12]. The results of this study showed that PYY and GLP-1 changed in the direction of appetite blunting, while ghrelin did not. One reason for this disagreement could be due to the high variability in ghrelin values in this study [12].

We found a trend for a decrease in acylated ghrelin immediately post-exercise (*p* = 0.014; 22% decrease), which is similar to previous studies in trained men [7,8,9,11,19,20,21,23,25,26]. In the studies examining male participants, both cycling and running at a range of intensities (64%–72% VO_2MAX_) and durations (45–105 min), resulted in significantly lower circulating acylated ghrelin post-exercise (~50%–90% decrease). However, Larson-Meyer *et al.* [12] found an increase in acylated ghrelin immediately post-exercise (50% increase) in trained females. This suggests that acylated ghrelin is responsive to varied conditions and modes of endurance exercise, duration, and moderate- and high-intensities, but the direction of this response is varied. Some of these differences may be attributed to subject fitness, pre-exercise meal composition and timing, and the timing of the hormone measurements [33]. None of these studies investigated differences in exercise intensity.

We found significant increases in PYY_3–36_ post-exercise exercise (*p* = 0.02; 9.5% increase). Others report post-exercise increases of 10%–92%, when examining the effect of exercise intensity on PYY in highly-trained male athletes (VO_2MAX_ ≥ 45.9 mL/kg/min) at intensities of 70%–85% VO_2MAX_ for durations of 40 to 90 min [8,10,13,21,23,25,29]. The only study investigating the effects of exercise on PYY in highly-trained women (VO_2MAX_ = 49.7 mL/kg/min) found results similar to ours. Larson-Meyer and colleagues [12] found significantly higher PYY after running for 60-min at 70% VO_2MAX_ (22% increase). One explanation for why our data are similar to the Larson-Meyer *et al.* [12] results for PYY, while they were different for ghrelin, is that the PYY data were less variable with all participants having similar responses.

Only two studies have examined the effect of exercise intensity on blood concentrations of PYY. Ueda and colleagues [10] found that circulating PYY was significantly higher after 30-min of cycling at 75% VO_2MAX_ than 50% VO_2MAX_ in active males (VO_2MAX_ = 45.9 mL/kg/min). A follow-up study by Deighton and colleagues [13] found similar results between 60-min cycling at 59.5% VO_2MAX_ and 10 × 4 min cycling at 85.8% VO_2MAX_ in highly-trained males (VO_2MAX_ = 52.4 mL/kg/min). They reported that circulating PYY_3–36_ was higher after the moderate-intensity trial (*p* value not given), but AUC was significantly higher during the hours after the high-intensity trial.

Based on these findings, we hypothesized that PYY would be higher after the HIE (85% VO_2MAX)_ compared to MIE (60% VO_2MAX_). Conversely, we found no differences in circulating PYY between exercise intensities. One reason for differences between trials may be due to the clamping of energy expenditure *vs.* time among the various studies. In the current study, we held energy expenditure constant (500 kcals or 2093 kJ) during the moderate and high intensity trials, whereas Ueda and colleagues [10] controlled for time and not energy expenditure. Thus, discrepancies between results may be due to differences in energy expenditure, rather than exercise intensity. In addition, our participants were highly-trained women (VO_2MAX_ = 55.2 mL/kg/min), whereas the participants in the Ueda *et al.* [10] study were active males (VO_2MAX_ = 45.9 mL/kg/min), but not as highly-trained. Based on a meta-analysis done by Schubert *et al.* [34] there is evidence to suggest that highly-trained individuals are more accustomed to the stress of exercise and therefore do not have as great a hormonal response as untrained individuals. In contrast to Deighton *et al.* [13], who used male participants, our trials were steady-state running, whereas the Deighton and colleagues included a moderate steady-state cycling bout (60-min, 59.5% VO_2MAX_) and a high-intensity intermittent cycling trial (4 × 10 min, 85.8% VO_2MAX_). The nature of the intermittent high-intensity trial may differ from our high-intensity steady state trial. Thus, because of the differing exercise protocols, it’s difficult to compare results between these studies.

Limited research has examined changes in GLP-1 due to exercise. Results from Ueda and colleagues [10] and Larson-Meyer and colleagues [12] reported significant increases in GLP-1 post-exercise. Similar to our results (*p* < 0.0001; 77% increase), both studies found elevated GLP-1 after 60-min running or walking at 70% VO_2MAX_ (78% increase) [12] and 30-min cycling at 50% or 75% VO_2MAX_ (23% increase) [10]. Similar to PYY, post-exercise circulating GLP-1 may be dependent on the energy expenditure of activity. Larson-Meyer and colleagues [12] found that circulating GLP-1 was elevated after 60-min running, but not after walking at the same relative intensity and duration. However, energy expenditure in the running trial was 37% greater than in the walking trial because of the higher VO2max in the runners compared to the walkers. In addition, there are a number of physiological differences between running and walking that might contribute to differences hormonal responses, such as great sympathetic nervous system activity, greater gastrointestinal motility, and body temperature [33].

Overall, our results are similar to other research examining appetite-regulating hormones after an acute bout of exercise in highly-trained males and females. Endurance exercise lasting 30–90 min at exercise intensities between 50% and 85% VO_2MAX_ alter appetite-regulating hormones (acylated ghrelin, PYY, and GLP-1) in the direction of appetite suppression [7,8,9,10,11,13,19,20,21,23,24,25,26,32]. A recent meta-analysis by Schubert and colleagues [35] found that studies involving exercise with greater energetic and mechanical demands, such as running, had greater appetite suppression. One speculation for this difference with running is that splanchnic blood flow is reduced during running, inhibiting ghrelin secretion [35]. Thus, the lack of consistency in results measuring the impact of exercise on appetite may be attributed to the different modes of exercise chosen. The impact of running on the gut may produce different results than cycling, due to less jarring of the stomach and less mechanical damage. Further, Kawano and colleagues [21] found that weight-bearing exercise (rope skipping) resulted in greater appetite suppression than non-load bearing (cycling) in men. Since many of the appetite-regulating hormones are released from the gut the mode of exercise may impact the concentration of circulating peptides post-exercise.

### 4.3. Appetite Ratings, VAS and Appetite Hormones

Studies examining the impact of exercise on ratings of appetite (VAS) in active highly active males are equivocal. While most studies report appetite suppression (30–90 min, 50%–85.8% VO_2MAX_) [7,8,9,10,11,13,20,21,25,28,36], others find no effect of exercise on appetite (34–90 min, 35%–75% VO_2MAX_) [22,23,26,30,37]. This lack of consistency in study results may be due to the limitation and sensitivity of the VAS. Although VAS is frequently used in exercise and appetite studies in active individuals, the original validation studies were done in healthy sedentary males [16].

Only two studies have investigated the influence of exercise on subjective appetite in highly-active women [12,38]. Both studies found a trend for appetite suppression post-exercise (37–65 min, 41%–70% VO_2MAX_) using the VAS assessment tool. Pomerleau and colleagues [38] found significantly lower ratings of appetite immediately post-exercise, but no impact of exercise intensity (41% *vs.* 70% VO_2MAX_) on appetite ratings. Similarly our study showed that exercise can suppress appetite, but was independent of exercises intensity.

Overall, we found a transient suppression of appetite that returned to baseline within 60-min post-exercise. This is similar to the findings in men [39]. Thus, exercise alone, independent of intensity, has the ability to suppress appetite, and potentially reduce food intake immediately post-exercise. We found no difference in ratings of appetite due to exercise intensity. Our findings are consistent with recent studies investigating the impact of moderate- and high-intensity exercise on ratings of appetite in highly-active males [10,13] and females [38]. Ueda and colleagues [10] found that ratings of appetite were suppressed post-exercise in highly-active men, but no differences between moderate (50% VO_2MAX_) and high-intensity (75% VO_2MAX_) exercise trials. Pomerleau and colleagues [38] found similar results in highly-active women; exercise suppressed appetite, but there were no differences between moderate- (40.9% VO_2MAX_) and high-intensity (69.4% VO_2MAX_). Conversely, Larson-Meyer and colleagues [12] found no effect of exercise on appetite. Lack of consistency in these studies may be due to the limitation of VAS in detecting small changes in appetite [16] or the various exercise protocols employed, such as total energy expended in the exercise session or participant level of training.

Overall, results from the blood and appetite ratings followed a similar pattern towards appetite suppression immediately post-exercise, with a return to baseline 60-min post-exercise. In general, most studies using highly-trained males have found similar results [7,8,9,10,11,13,20,21,25]. Only Larson-Meyer *et al.* [12] report blood appetite hormones and appetite ratings in highly-active females. They found increased PYY and GLP-1 post-exercise, indicating appetite suppression, but also increased acylated ghrelin (appetite stimulation), and increased appetite ratings after 60-min of running at 70% VO_2MAX_. Thus, our study results were more similar to those reported in highly-trained males.

### 4.4. Strengths and Limitations

All participants were highly-trained women, accustomed to regular exercise at high intensities. The exercise intensities selected for MIE and HIE trials also mimicked a typical training regime of an endurance athlete, 60% VO_2MAX_ representing an easy distance session, and 85% VO_2MAX_ representing a hard intensity session. Since many high level athletes regularly include moderate and high-intensity exercise as part of normal training plan, the results of this study are applicable to real life.

Second, energy expenditure was held constant between trials (500 kcal/trial or 2093 kJ/trial) so that differences between exercise intensities and not energy expenditure could be assessed. Only two other studies have controlled for energy expenditure in highly-trained participants. Pomerleau and colleagues [38] used 350 kcal walking at 40% or 70% VO_2MAX_ in highly-trained females, and Imbeault and colleagues [37] used 490 kcal walking at 35% VO_2MAX_ or running at 75% VO_2MAX_ in highly-trained males. The other studies either set a similar time or paid no attention to clamping time or energy expenditure, making comparisons between studies difficult.

Lastly, diet and physical activity were monitored before each trial to hold these variables constant. Participants were required to refrain from activity for at least 24-h before each trial and to keep a food and physical activity logs for 48-h before each trial. Although we did not manipulate diet, participants were asked to keep 48-h intakes similar between trials. Compliance was checked before each trial, with all participants being compliant.

We did not measure energy intake for an extended period of time after exercise to determine if energy compensation occurred regardless of exercise intensity and similar energy expenditures. Typically athletes report feeling less hunger after higher intensity exercise [40]. We matched energy expenditure, so would expect our highly active, weight stable participants to adequately compensate. In this study, each subject served as their own control, thus, we did not have a rest only control group. We could not control for menstrual cycle and also administer the trials approximately 1-week apart. Research shows that appetite-hormones do not change significantly throughout the menstrual cycle [41]. Finally, a larger sample size would have assured that our study was powered to see true changes in exercise intensities if they did exist.

## 5. Conclusions

An acute bout of exercise has the ability to transiently suppress appetite-regulatory hormones immediately post-exercise in highly-trained women. This suppression occurs independent of exercise intensity. For endurance female athletes who are trying to optimize nutrition for performance and maintenance of lean tissue, refueling after exercise is an important nutrition strategy. Thus, female athletes who regularly train for endurance competitions can expect to experience appetite suppression immediately post-exercise. Unfortunately, this suppression of appetite comes during the key window of time (e.g., 30–40 min post-exercise) when the body is more metabolically primed for anabolic processes if fuel and nutrients are received to facilitate recovery. If refueling does not occur immediately post-exercise, recovery may be delayed or incomplete. This can impact subsequent exercise sessions, resulting in lower quality workouts and greater fatigue. Over time inadequate refueling can lead to poor athletic performance, overtraining, or injury. Female athletes need to be conscious of exercise appetite suppress and select foods or calorie-containing beverages they will consume even when they do not feel hungry. Defining post-exercise nutrition recommendations for highly-trained women will help optimize training and recovery and improve subsequent endurance performance.

## Figures and Tables

**Table 1:** Experimental trials.

		Pre-Exercise	Exercise EE = 500 kcals (2093 kJ)	Post-Exercise (Rest)				
	**48-h Pre-Visit**	**Baseline**	**HIE or MIE**	**0-min**	**20-min**	**40-min**	**60-min**	**24-h Post Visit (Before Meals, Pre-and Post-Exercise)**
Gut Hormones		•		•	•	•	•	
VAS		•		•	•	•	•	
Food Log	•							•
PA Log	•							•

• Indicates when data were collected. EE = energy expenditure, VAS = visual analog scale, HIE = high-intensity exercise, MIE = moderate intensity exercise, EE = energy expenditure, PA = physical activity. Exercise Trials Format: MIE or HIE. Participants Ran on a Motorized Treadmill until Exercise Energy Expenditure (EE) Equaled 500 kcal (2093 kJ). Exercise Was Performed at Least 4-h Post-Prandial and Testing Order was Randomized with a 7-day Washout Period between Trials.

**Table 2:** Subject demographics.

**Age (years)**	31.1 ± 6.7
**Height (cm)**	166 ± 7
**Weight (kg)**	58.4 ± 6.4
**BMI (kg/m^2^)**	21.1 ± 1.7
**Body Fat (%)**	14.6 ± 2.9
**VO_2MAX_ (mL/kg/min)**	55.2 ± 4.3

Data are means ± SD. BMI (kg/m^2^) = body mass index, Body fat percentage via 7-site skin fold, VO_2MAX_ = maximal oxygen uptake via open-circuit spirometry while running on a motorized treadmill.

**Table 3:** Moderate-Intensity Exercise (MIE) and High-Intensity Exercise (HIE) Trials Data Summary.

	Moderate Intensity Exercise (MIE) (60% VO_2MAX_)	High Intensity Exercise (HIE) (85% VO_2MAX_)
Duration (min)	45.7 ± 10.8	33.6 + 5.6
Speed (m/min)	157.1 ± 17.3	199.7 ± 21.3
Grade (%)	0.9 ± 1.1	2.6 ± 2.7
VO_2_ (mL/kg/min)	36.4 ± 3.0	47.8 ± 3.7
Heart Rate (BPM)	139 ± 11	169 ± 8

Data are means ± SD. VO_2_ = oxygen uptake, BPM = beats per minute.

## Abbreviations

The following abbreviations are used in this manuscript:

MDPI	Multidisciplinary Digital Publishing Institute
DOAJ	Directory of open access journals
TLA	Three letter acronym
LD	linear dichroism
